# Hörgeräteversorgung und Qualitätssicherung

**DOI:** 10.1007/s00106-025-01626-z

**Published:** 2025-04-03

**Authors:** Annette Limberger

**Affiliations:** 1 Studiengang Audiologie und Hörakustik, Hochschule Aalen, Beethovenstr. 1, 73430 Aalen, Deutschland; 2 Klinik für Hals-Nasen-Ohrenheilkunde, Kopf-Hals-Chirurgie, SLK-Kliniken Heilbronn, Am Gesundbrunnen 20–26, 74078 Heilbronn, Deutschland

**Keywords:** Tragbare elektronische Geräte, Sensorische Hilfssysteme, Hörverlust, Sprachverständlichkeit, Reliabilität und Validität, Wearable electronic devices, Sensory aids, Hearing loss, Speech intelligibility, Reliability and validity

## Abstract

Etwa 17,1 % der Bevölkerung in Deutschland sind schwerhörig, das sind etwa 12,1 Mio., allerdings haben davon nur etwa 3,7 Mio. ein Hörgerät. Ein nicht ausreichend versorgter Hörverlust hat nicht nur enorme Auswirkungen auf die Lebensqualität, sondern verursacht auch noch enorm hohe Kosten, so veranschlagte eine Metastudie die Kosten der unbehandelten Hörverluste für ganz Europa mit 216 Mrd. € jedes Jahr. Dabei kann eine Hörsystemversorgung die Gesundheit und v. a. Lebensqualität deutlich verbessern. Ein unbehandelter Hörverlust kann jedoch zu sozialer Isolation, Depression und vorzeitigem kognitivem Abbau führen, während eine adäquate Versorgung diesen Folgen vorbeugt. Die vorliegende Übersicht gibt einen Überblick über die Indikationen und den Ablauf einer Hörsystemversorgung und wie diese im Sinne der Qualitätssicherung überprüft werden sollte, um Menschen mit einer Schwerhörigkeit bestmöglich zu versorgen.

## Abstract (en)

Approximately 17.1% of the German population, so around 12.1 million people, suffer from hearing loss; however, only around 3.7 million of them have a hearing aid. Inadequately treated hearing loss not only has an enormous impact on quality of life but also causes extremely high costs. A meta-analysis of many European countries estimated that untreated hearing loss costs Europe 216 billion euros every year. Hearing system provision can significantly improve health and, above all, quality of life, while untreated hearing loss can lead to social isolation, depression, and premature cognitive decline—consequences that adequate provision can prevent. This overview provides a summary of the indications for and provision of a hearing system and discusses how these should be checked in terms of quality assurance in order to provide the best possible care to people with hearing loss.

## Vorteile der Hörgeräteversorgung

In Deutschland kann man, hochgerechnet aus der Untersuchung durch von Gabelenz et al., davon ausgehen, dass etwa 17,1 % der Bevölkerung einen Hörverlust von mindestens 25 dB haben [11]. Allerdings ist davon auszugehen, dass nur ein Teil dieser Menschen, etwa 3,7 Mio., mit Hörgeräten versorgt ist [16]. Ein nicht behandelter Hörverlust kann sich nicht nur besonders ungünstig auf die Lebensqualität auswirken, sondern verursacht auch noch hohe Kosten. Dies wurde in einer Metaanalyse aus mehreren europäischen Staaten ersichtlich, so belaufen sich diese Kosten für Europa auf etwa 216 Mrd. € pro Jahr, heruntergebrochen auf Deutschland sind dies etwa 39 Mrd. € pro Jahr [28]. Weitere negative Auswirkungen eines nicht versorgten Hörverlusts, nicht nur auf die Volkswirtschaft, zeigen sich in einem sozialen Rückzug, Depression und einem vorzeitigen kognitiven Abbau. Dem kann eine Hörsystemversorgung vorbeugen, v. a. kann sie durch eine Verbesserung der Kommunikation die Lebensqualität deutlich steigern [5].

**Abb. 1:**
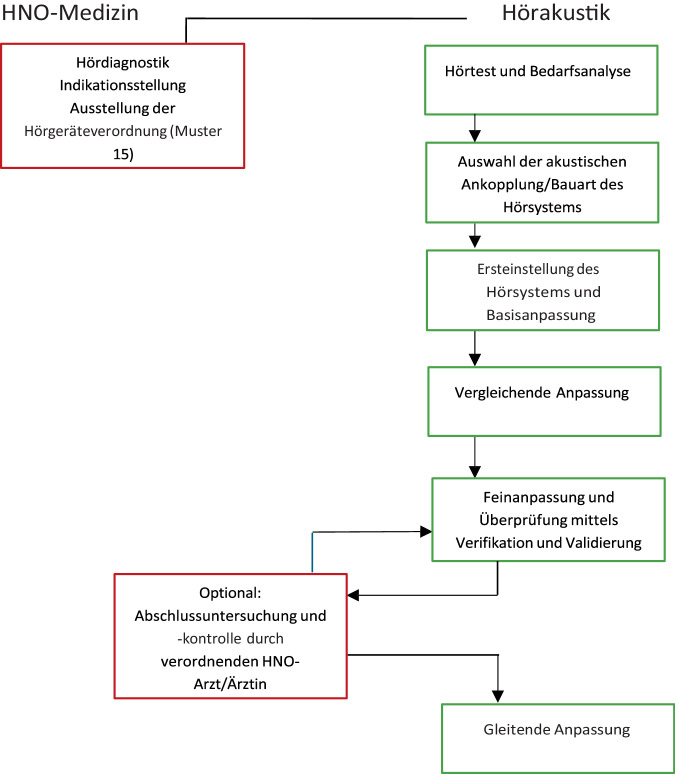
Ablauf einer Hörgeräteversorgung im Rahmen der gesetzlichen Versicherung

Daher ist eine gute Hörsystemversorgung die Grundlage für eine verbesserte Kommunikation und daher Teilhabe am gesellschaftlichen Leben. Im Folgenden wird die Hörgeräteversorgung und deren Überprüfung im Sinne einer Qualitätssicherung dargestellt. Der schematische Ablauf einer Hörsystemversorgung ist in Abb. 1 illustriert und wird im Folgenden erläutert. Die Begriffe Hörgerät und Hörsystem werden im Rahmen dieses Textes z. T. synonym verwendet, wobei der Begriff Hörgerät eher nur das Gerät als solches meint, mit dem Begriff Hörsystem wird das Hörgerät mit entsprechender Otoplastik und ggf. weiterem Zubehör bezeichnet.

## Hilfsmittel-Richtlinie

Grundlage der Hörgeräteversorgung im Rahmen der vertragsärztlichen Versorgung ist die Hilfsmittel-Richtlinie (HilfsM-RL) des Gemeinsamen Bundesausschusses (G-BA), die zuletzt im Jahr 2021 erneuert wurde [13].

Hier ist festgelegt ab welchem Hörverlustwert eine Hörgeräteversorgung zulasten der Gesetzlichen Krankenversicherung (GKV) abgerechnet werden kann, aber auch welcher Versorgungserfolg mit einer entsprechenden Versorgung erzielt werden sollte. Im Bereich der kassenärztlichen Versorgung sind Qualitätssicherungsmaßnahmen nach § 135 Abs. 2 SGB V durch ergänzende Ausführungen reglementiert [19].

Vor der Verordnung einer Hörhilfe wird eine audiologische Differenzialdiagnostik mithilfe der Ton- und Sprachaudiometrie, Impedanzaudiometrie, Bestimmung der Unbehaglichkeitsschwelle, sowie, falls notwendig, weiterer diagnostischer Verfahren wie akustisch evozierter Potenziale (BERA oder CERA) und otoakustischer Emissionen (TEOAE und DPOAE) durchgeführt.

Eine beidohrige Hörsystemversorgung zulasten der GKV ist dann indiziert, wenn der tonaudiometrische Hörverlust auf der besseren Seite in einer der Frequenzen zwischen 500 und 4000 Hz 30 dB_HL_ oder mehr beträgt und die Sprachverständlichkeit unter Verwendung des Freiburger Einsilbertests bei 65 dB_SPL_ nicht mehr als 80 % beträgt [13]. Diese Bedingungen gelten auch für eine einseitige Versorgung für das schlechtere Ohr [13].

Vor der Empfehlung für ein bestimmtes Hörsystem sollte eine ausführliche Bedarfsanamnese und -analyse durchgeführt werden. Dies beinhaltet zu erfragen, in welchen Hörsituationen Probleme auftreten und wie sich diese äußern. Dies ist besonders hilfreich und wichtig bei Menschen, die noch berufstätig sind, hier gilt es zu erfahren, ob sie häufig wechselnde Hörumgebungen haben, gibt es Störgeräusche, oder handelt es sich um einen Lärmarbeitsplatz u. a. m.

Bei der Auswahl des Hörsystems sind aus audiologischer Sicht v. a. die akustische Ankopplung und Überlegungen zu den Eigenschaften des Hörsystems wichtig. Die Bauart des Hörsystems ist eng verknüpft mit der akustischen Ankopplung.

### Bauart

**Abb. 2:**
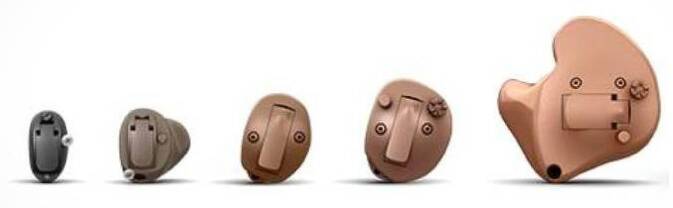
Im-Ohr-Hörsysteme. *Von links nach rechts *Invisible-in-the-Canal(IIC)-, Completely-in-the-Canal(CIC)-, Im-Ohr(IO)-, Halbconcha- und Concha-System (© Oticon, Kopenhagen, Dänemark)

Unterschieden werden Im-Ohr-Hörgeräte (IO) von Hinter-dem-Ohr-Geräten (HdO). IO-Geräte werden eingeteilt in Invisible-in the-Canal-Geräte(IIC)-, Completely-in-the-Canal(CIC)-, In-the-Ear(ITE)-, Halbconcha- und Concha-Geräte (Abb. 2). Hierbei sitzen die IIC-Geräte im knöchernen Gehörgang, direkt vor dem Trommelfell, CIC-Geräte im knorpeligen Gehörgang bis zum Isthmus, dem Übergang zum knöchernen Anteil, ITE-Geräte schließen i. d. R. mit dem Tragus ab und sind gerade von außen sichtbar, während IIC- und CIC-Geräte von außen praktisch unsichtbar sind, sichtbar ist nur ein kleiner Nylonfaden, an dem das System aus dem Gehörgang wieder entfernt werden kann. Es gibt ein IIC-System, welches dazu gedacht ist, über Monate im äußeren Gehörgang zu verbleiben, wobei hier bereits die Entstehung eines Cholesteatoms des äußeren Gehörgangs beschrieben wurde [29]. Weitere Bauarten wie das Halbconcha- und Concha-Gerät füllen die Concha ganz oder teilweise aus. Die Hauptlimitation der meisten Im-Ohr-Systeme ist die Verstärkung, da hier Mikrofon und Hörer auf engstem Raum liegen und es daher sehr schnell zu Rückkopplungen kommen kann bzw. durch das Rückkopplungsmanagement der Hörsysteme die Verstärkung gedrosselt wird. Ein weiterer Grund, der zum Ausschluss eines Im-Ohr-Systems führt, ist häufig der mangelnde Platz innerhalb des äußeren Gehörgangs, sodass dies dann zu einer größeren Bauart führen würde, die für die Betroffenen kosmetisch nicht mehr so ansprechend wäre. Ein weiteres Problem kann das Empfinden der eigenen Stimme sein. Durch den Verschluss des äußeren Gehörgangs kann es zu einem Okklusionseffekt kommen, der zu einer veränderten Empfindung der eigenen Stimme führt, die zur schlechteren Akzeptanz von Hörsystemen führen kann [23]. Vorteile, der IO-Systeme sind die natürlichere Schallaufnahme im Bereich des äußeren Gehörgangs und damit Ausnutzung der Richtungshörwirkung der Ohrmuschel sowie die direkte Schallabgabe vor dem Trommelfell.

**Abb. 3:**
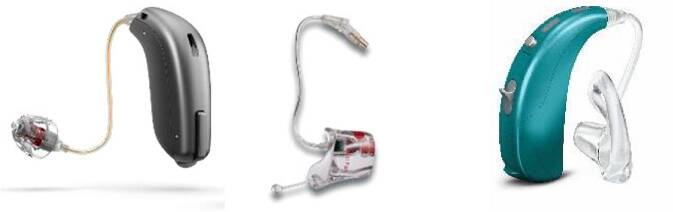
Hinter-dem-Ohr-Hörsysteme. *Von links nach rechts*: sog. Receiver-in-the-Ear(RITE)- oder Receiver-in-the-Canal(RIC)-System mit Schirmchen, Hörer mit individueller Otoplastik, Hinter-dem-Ohr(HdO)-System mit Otoplastik (©Audia-Akustik und Welttag des Hörens)

Die HdO-Geräte lassen sich in 2 Gruppen einteilen, die klassischen Geräte mit Schallzuführung in den Gehörgang durch einen Schallschlauch und i. d. R. einer individuellen Otoplastik sowie sog. Receiver-in-the-Canal(RIC)- oder Receiver-in-the-Ear(RITE)-Geräte, bei denen der Hörer (Lautsprecher) direkt im Gehörgang sitzt (Abb. 3). Auch dieser Hörer sollte i. d. R. in eine individuelle Otoplastik eingebettet sein (Abb. 3, 2. v. l.). Das Hörsystem selbst wird hinter dem Ohr getragen. Diese sind besonders gut zur Versorgung von Hochtonhörverlusten geeignet, da ein Schallschlauch wegfällt und damit hohe Frequenzen besser übertragen werden.

Unter den Personen, die in Deutschland mit Hörsystemen versorgt sind, haben knapp 20 % ein IO-Gerät, rund 78 % ein HdO-Gerät; von diesen wiederum sind 47 % mit einem RIC-Gerät versorgt [9].

### Otoplastik

Zur Herstellung einer individuellen Otoplastik wird eine Abformung des äußeren Ohrs vorgenommen, diese wird inzwischen meist eingescannt und mittels 3‑D-Druck hergestellt. Gängige Materialien sind Polymethylmethacrylat (Acryl), Lichtpolymerisat, Polyurethan (Handelsname THERMOtec, Fa. DETAX GmbH, Ettlingen) und Silikon. Acryl-Otoplastiken sind gut zu bearbeiten und günstig in der Herstellung, haben jedoch den Nachteil, dass sie durch einen hohen Restmonomergehalt zu Hautirritationen und Allergien führen können, daher werden sie mit einer Schicht Lichtpolymerisat überzogen, dies müsste nach jeder Bearbeitung erneuert werden. Lichtpolymerisat ist auch seit Langem in der Zahnmedizin bekannt, und durch die Aushärtung unter UV-Licht ist dieses Material durch einen sehr hohen Polymerisationsgrad gut hautverträglich. Polyurethan, ein vollvernetzendes Polymer, wird unter dem Namen THERMOtec gehandelt. Vorteil des Materials ist außerdem die Durchlässigkeit für Feuchtigkeit und die Änderung der Elastizität mit der Temperatur. Im Ohr ist das Material eher weich, im kalten Zustand eher hart und kann v. a. im gekühlten Zustand gut bearbeitet werden. Silikonmaterialien werden häufig für stark verstärkende Hörsysteme verwendet, da diese das Ohr gut abdichten und so die Neigung zu Rückkopplung minimiert wird, durch die guten Methoden der Rückkopplungsauslöschung ist dies heute nicht mehr so relevant. Ein weiterer wichtiger Einsatz findet sich in der Pädaudiologie, Kinder werden praktisch immer mit Silikonotoplastiken versorgt, die es in unterschiedlichen Härtegraden gibt. Für Babys und Kleinstkinder sind Shore-Härten von 15–25 zweckmäßig, da diese sehr weich bzw. weich sind.

Der durchgezogene Schallschlauch hat in den meisten Fällen einen Innendurchmesser von 2 mm, der Außendurchmesser kann durchaus variieren. Da der Schallschlauch akustisch durchlässig ist, wird bei hochgradigen Hörverlusten eine Wandstärke von 2 mm gewählt, somit liegt der Außendurchmesser bei 4 mm. Der Schallschlauch selbst ist, akustisch gesehen, zudem ein Tiefpass, d. h. tiefere Frequenzen werden gut übertragen, höhere weniger gut, daher sollte insbesondere bei Hochtonhörverlusten ein dünner Schallschlauch (sog. Slim-Tube, Innendurchmesser etwa 1 mm) vermieden werden.

**Abb. 4:**
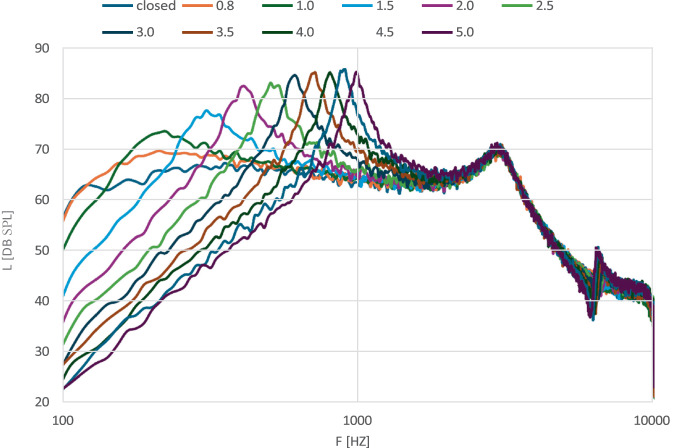
Verschiedene Ventgrößen, gemessen mit der Messbox (ACAM 5) und einem breitbandigen Rauschen von 65 dB_SPL_. Mit zunehmender Ventgröße in mm auftretende Reduktion der Verstärkung im tiefen Frequenzbereich < 800 Hz, im höherfrequenten Bereich Resonanzspitze entstehend, deren Frequenz ebenfalls vom Ventdurchmesser abhängig

Zusatzbohrungen (Vent) werden verwendet, um einerseits bei gutem Hörvermögen im Tieftonbereich (< 1000 Hz) den Okklusionseffekt zu mindern, andererseits, um eine Belüftung des Gehörgangs zu gewährleisten. Unterhalb von einem Millimeter haben Zusatzbohrungen praktisch keinen Einfluss auf den Frequenzgang des Hörsystems und werden daher oft als Belüftungsbohrung bezeichnet. Das Ausmaß der Wirkung auf den Frequenzgang ist abhängig von Länge und Durchmesser des Vents, der sog. akustischen Masse (Ma) in Henry (nach Beranek) [8]. In Abb. 4 wurden verschiedene Ventgrößen, gemessen mit der Messbox (ACAM 5, Fa. Acousticon, Reinheim, Deutschland) und einem breitbandigen Rauschen von 65 dB_SPL_. Mit zunehmender Ventgröße in mm geht Verstärkung im tiefen Frequenzbereich < 800 Hz verloren, im höherfrequenten Bereich entsteht eine Resonanzspitze, deren Frequenz ebenfalls vom Ventdurchmesser abhängig ist.

Mithilfe dieser Zusatzbohrung kann so der Frequenzgang im Tieftonbereich beeinflusst werden, was v. a. bei Hochtonhörverlusten zum Einsatz kommt, jedoch muss auch bedacht werden, dass durch eine solche Zusatzbohrung auch Direktschall von außen an das Ohr gelangen kann, der damit z. B. Features wie z. B. Störgeräuschreduktion negativ beeinflussen kann, ebenso eine Richtcharakteristik der Mikrofone. Das Credo sollte daher sein: So geschlossen wie möglich, so offen wie nötig. Inzwischen werden neuere Otoplastikformen verwendet, die unter dem Namen Nugget-Otoplastik bekannt wurde, hierbei wird eine großzügige Aussparung im vorderen unteren Bereich des Gehörgangs gelassen, die v. a. das Kiefergelenkköpfchen ausspart [6]. Hier kann der Okklusionseffekt bei geschlossener Otoplastik praktisch vermieden werden [6]. Von sog. Domes oder Schirmchen ist abzuraten, da diese keine fest definierte Lage im äußeren Gehörgang haben und sich so die Übertragungseigenschaften ständig verändern können.

## Auswahl des Hörsystems

Das Hörsystem richtet sich nach den Hör-Anforderungen. Bei der Versorgung zulasten der GKV ist momentan eine Mindestausstattung für die zuzahlungsfreie Versorgung vorgeschrieben. Hierzu gehören derzeit [30]:

- Digitaltechnik
- Mehrkanaligkeit (mindestens 6 Frequenzkanäle)
- Adaptive Rückkopplungs- und Störschallunterdrückung
- Mindestens 3 Hörprogramme
- Adaptive omnidirektionale und gerichtete Schallaufnahme/Mehrmikrofontechnik (adaptive Mehrmikrofontechnik, sofern nicht vom Versicherten aus kosmetischen Gründen eine IO-Versorgung ohne Mehrmikrofontechnik gewünscht wird oder die Schallaufnahme im Gehörgang erfolgt)

Im Übrigen sollte das Hörsystem nach den Höranforderungen ausgewählt werden. Wobei zusätzliche Features zum Einsatz kommen können, wie z. B. die binaurale Interaktion der Hörsysteme, die kurze Audiosequenzen gegenseitig austauschen. Dadurch kann eine bessere Richtungshörwirkung erzielt werden, da u. a. die interaurale Pegeldifferenz beibehalten wird und so das Sprachsignal, so es von einer Seite kommt, auch von dieser Seite wahrgenommen wird. Bei Hörsystemen, die getrennt voneinander arbeiten, fällt diese interaurale Pegeldifferenz („interaural level difference“, ILD) weg, und eine räumliche Analyse kann z. B. nur durch einen Laufzeitunterschied („interaural time difference“, ITD) durchgeführt werden, was die Richtungshörwirkung erschwert. Auch können so verbesserte Richtungshörwirkungen und damit Störgeräuschreduktionsalgorithmen realisiert werden, die zu einem verbesserten Sprachverstehen im Störgeräusch führen [7, 26, 31]. Zusätzliche Möglichkeiten können aber auch die Bluetooth-Anbindungen sein, die eine Bedienung oder auch Programmierung der Hörsysteme über Apps zulassen.

## Anpassung der Hörsysteme

**Abb. 5:**
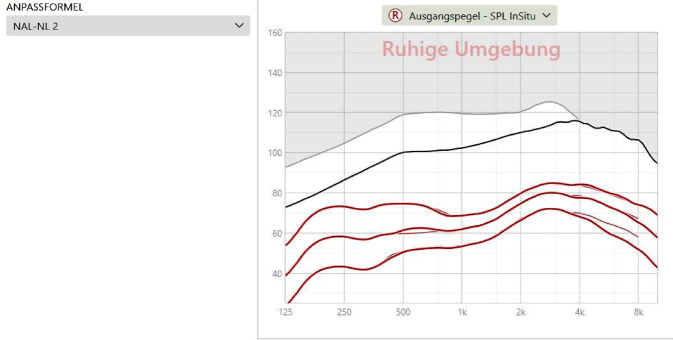
Einstellung des Hörsystems mithilfe einer Herstellersoftware, hier beispielhaft für eine rechte Seite. Gewählte Anpassformel: NAL-NL2. In der *Grafik rechts *die (berechneten!) Ausgangsschalldruckpegel für leise, mittlere und laute Eingangsschalldruckpegel (*von unten nach oben*). *Dickere Linie *die die Hörgeräteeinstellung simulierende Kurve, *dünnere Linie *die Zielkurve der gewählten Anpassformel. *Schwarze Kurve* maximaler Ausgangsschalldruckpegel. *Grauer Bereich* das Limit des angeschlossenen Hörsystems. Anhand dieser Darstellung offenbar Ausgangsschalldruckpegel des Hörsystems perfekt auf den Zielkurven liegend

Für eine erste Einstellung der Hörsysteme bieten alle Hersteller eine eigene Fitting-Software. In diese Software sollten, neben den persönlichen Daten, die Daten zum Hörverlust, aber v. a. auch zur akustischen Ankopplung eingegeben werden. Dadurch kann die Software näherungsweise die Einstellung der Hörsysteme berechnen und simulieren. Zur Einstellung selbst wird eine Anpassformel ausgewählt, entweder die herstellereigene Formel oder eine der generischen Formeln wie z. B. NAL-NL1 oder 2 bzw. DSL v 5.0 (Abb. 5).

NAL-NL1 ist eine Weiterentwicklung der Vorgängerformeln NAL und NAL-R(P) aus den National Acoustic Laboratories (NAL) in Australien. Die Grundidee, die der Anpassformel zugrunde liegt, ist, den sog. Speech Intelligibility Index (SII) ein rechnerisches Verfahren zur Vorhersage der Sprachverstehensquote, so optimal wie möglich zu erreichen. Die Formel nach NAL-NL1 („non-linear version 1“) berechnet für jeden beliebigen Hörverlust die Verstärkung in verschiedenen Frequenzbändern und für verschiedene Eingangspegel, den optimalen SII und eine Lautheitsempfindung, die normal ist, oder etwas darunter liegt [2]. Für die Formel nach NAL-NL2 („non-linear version 2“) wurden einige Veränderungen eingeführt, die v. a. sog. „dead regions“ auf der Cochlea berücksichtigen und hier weniger Verstärkung anbieten [20, 21].

Primär für die Hörgeräteversorgung von Kindern wurde die Formel nach dem „desired sensation level“ (DSL) entwickelt. Die derzeit aktuelle Version ist DSL v5 [27]. Die Verstärkung wird für verschiedene Eingangspegel so gewählt, dass alles, auch leise Eingangspegel, gehört werden, es aber nie zu laut wird. Auch diese Anpassformel ist für jeden beliebigen Hörverlust wählbar, im Vergleich zu NAL-NL1 oder 2 deutlich weniger kompressiv und bietet gerade in den hohen Frequenzen mehr Verstärkung.

Die herstellereigenen Anpassalgorithmen sind meist eine Mischung aus den beiden genannten Formeln, wobei die Verstärkung, insbesondere für leise Eingangspegel, zu Beginn der Anpassung häufig stark reduziert ist, um zunächst eine große Akzeptanz der Hörsysteme zu erreichen. Über die Zeit sollte die Verstärkung jedoch an den Hörverlust angepasst werden im Sinne einer Akklimatisierung.

Mithilfe der vorhandenen Formeln kann das Hörsystem zunächst annähernd gut eingestellt werden. So kann schnell auch eine vergleichende Anpassung mit verschiedenen Hörsystemen und Technikstufen durchgeführt werden.

## Verifikation

**Abb. 6:**
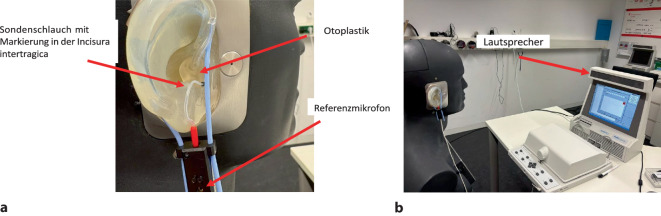
**a**,**b** In-situ-Messung oder Real-Ear-Measurement (REM). Testperson im geeigneten Abstand (meist etwa 40–60 cm, s. Herstellerangaben) vor dem Lautsprecher der Messanlage sitzend. **a** In den äußeren Gehörgang zuvor kalibrierter Sondenschlauch bis etwa 5 mm vor das Trommelfell eingeführt. Anschließend Hörsystem im Ohr platziert, ohne Veränderung des Sondenschlauchs (zur Hilfe kleine schwarze Markierung vorhanden). Einschalten des Hörsystems. **b** Abspielen des Internationalen Sprachtest(IST)-Signals mit einem bestimmten Eingangspegel aus dem Lautsprecher, Abgleich des Pegels am Referenzmikrofon und im Gehörgang vor dem Trommelfell Messung des Ausgangsschalldruckpegels

Um die Einstellung der Hörsysteme tatsächlich zu überprüfen ist eine In-situ-Messung notwendig. Dabei wird ein Sondenschlauch, der mit einem Mikrofon verbunden ist und der zuvor kalibriert wurde, vor das Trommelfell gelegt, die Otoplastik mit dem Hörsystem wird ebenfalls aufgesetzt (Abb. 6a), und es wird eine Messung des Ausgangsschalldruckpegels durchgeführt (sog. SPL-o-gramm), Abb. 6b. Diese Messung wird auch als Real-Ear-Aided-Response (REAR) bezeichnet [32].

Das Eingangssignal besteht aus dem Internationalen Sprachtest-Signal (ISTS), welches von der European Hearing Instrument Manufacturers Association (EHIMA) entwickelt wurde, um Hörgeräte international mit dem gleichen Sprachsignal zu messen. Das Signal besteht aus 6 verschiedenen Sprachen (Deutsch, Englisch, Französisch, Spanisch, Arabisch und Mandarin). Von den Sprecherinnen wurde jeweils der Text der Fabel „Der Nordwind und die Sonne“ gelesen. Die Sprachaufnahmen wurden in kleine Sequenzen geschnitten und diese zufällig aneinandergereiht [15]. Nun werden verschiedene Eingangspegel gewählt, häufig 50 dB für leise, 65 dB für mittlere und 80 dB für laute Sprache. Ausgegeben werden die Perzentilenkurven des Langzeitsprachspektrums meistens für das 30., das 60. und das 99. Perzentil für jedes Eingangssignal. Zur Messung des maximalen Ausgangsschalldruckpegels („maximum pressure output“, MPO) wird ein schmalbandiges Signal, z. B. ein Sinuston oder Sinussweep mit einem Eingangspegel von 90 dB verwendet (z. B. EUHA-MPO-Signal, Europäische Union der Hörakustiker e. V.) [10].

Hat man nun die Hörsysteme mit einer der generischen Formeln angepasst, so kann diese Formel auch im Messsystem ausgewählt werden, und es erscheinen die Zielkurven für die Ausgangsschalldruckpegel für den eingegebenen Hörverlust. So könnte nun das Hörsystem leicht an diese Zielkurven noch angepasst werden, dieser Prozess wird auch „fit-to-target“ genannt. Doch auch wenn die herstellereigene Anpassformel verwendet wurde, kann mithilfe der In-situ-Messung (Abb. 6 und 7) nachgewiesen werden, dass die Ausgangsschalldruckpegel für verschiedene Eingangsschalldruckpegel zum einen über der Hörschwelle liegen und damit die verschiedenen Laute auch gehört werden können, und zum anderen kann durch den MPO nachgemessen werden, dass die Unbehaglichkeitsschwelle nicht erreicht wird [32].

**Abb. 7:**
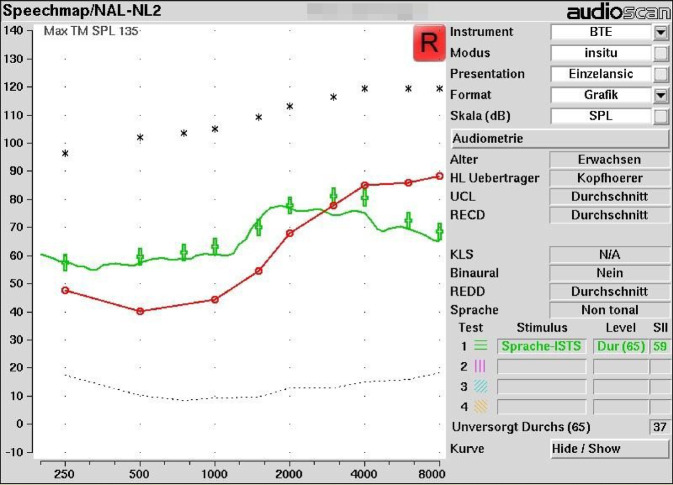
SPL-o-gramm oder auch In-situ-Messung, gemessen mit der Verifit-Software (Fa. Audioscan, A Division of Etymotic Design Inc., Dorchester, Ontario, Canada). Identischer Hörverlust wie für die Einstellung in der Software eingegeben. Kurven *von unten nach oben*: *gestrichelte Linie *Hörschwelle des Normalhörenden in dB_SPL_, *rot *das Audiogramm für die rechte Seite in SPL-Darstellung, *grün *der Ausgangsschalldruckpegel (60. Perzentile) für mittlere (65 dB) Eingangspegel des ISTS (s. *Tabelle rechts*), *Grüne Kreuze *Zielkurve nach der Anpassformel NAL-NL2. *Sternchen *geschätzte Unbehaglichkeitsschwelle, ebenfalls aufgeführt in der *Tabelle rechts:* der SII (Speech Intelligibility Index). Versorgt (in diesem Beispiel) ein SII von 59 erreicht, unversorgt von 37. Ab einer Frequenz von etwa 3 kHz der gemessene Ausgangsschalldruckpegel unterhalb der Hörschwelle liegend, und damit nicht wahrnehmbar

Konnte nun die adäquate Verstärkung am Trommelfell sichergestellt werden, kann nun die Validierung der Anpassung erfolgen. Im vorliegenden Beispiel der Abb. 7 wurde die adäquate Verstärkung v. a. im Hochtonbereich ab 3 kHz (Abb. 6) für mittlere Eingangspegel nicht erreicht. Der Ausgangsschalldruckpegel für mittlere Eingangsschalldruckpegel liegt unterhalb der Hörschwelle, und hohe Frequenzen über 3 kHz sind damit für den Menschen mit diesem Hörverlust nicht hörbar. Hier müsste nun über die Hersteller-Software das Hörsystem erst optimiert werden, bevor die Validierung erfolgen kann. Sollte sich kein Hörsystem finden, mit dem der Hochtonbereich suffizient verstärkt werden kann, sollte z. B. ein anderer Hersteller gewählt werden oder eine Frequenzverschiebung eingesetzt werden, bei der der nichthörbare Hochtonbereich in einen tieferfrequenten Bereich verschoben wird.

## Validierung

Zur Überprüfung des Anpasserfolgs stehen verschiedene Möglichkeiten zur Verfügung, wobei die Messung des Sprachverstehens in Ruhe und im Störgeräusch eine zentrale Rolle einnimmt. Gemäß der HilfsM-RL kann das Hörhilfenversorgungs-Ergebnis mit dem Freiburger Einsilbertest im freien Schallfeld überprüft werden, hierbei sollte eine Steigerung um 20 % erzielt werden, sofern ohne Hörgeräte noch ein Einsilberverstehen ermittelbar war; ist dies nicht der Fall, soll dem maximalen Einsilberverstehen möglichst nahe gekommen werden [13]. Es ist auch eine Überprüfung im Störgeräusch möglich, dabei soll der Sprachschallpegel 65 dB und der Störgeräuschpegel 60 dB betragen, der Gewinn mit den Hörgeräten soll mindestens 10 % betragen, bei Verwendung von 2 Testlisten je Testung [13]. Hier ist anzumerken, dass aus der HilfsM-RL nicht hervorgeht, in welcher räumlichen Anordnung sowohl Signal als auch Störgeräusch kommen sollte, dies kann v. a. beim Vergleich von Messungen aus der HNO-ärztlichen Praxis und der Messung im Hörakustikgeschäft zu großen Unterschieden führen [17]. Daher sollte auf jeden Fall die räumliche Anordnung notiert werden, oder es findet sich ein Konsens über die räumliche Anordnung, die von Kerner et al. mit Signal von vorne (S0) und Störgeräusch von ± 45° (N ± 45) vorgeschlagen wird [22].

Es wird auch für den Matrix-Test, den Oldenburger Satztest, sowie für den Göttinger Satztest ein bestimmtes Maß an Verbesserung gefordert, so ist der Erfolg belegt, wenn bei einem Pegel von 45 dB sprachsimulierendem Rauschen der Signal-Rausch-Abstand („signal to noise ratio“, SNR) mit Hörgeräten im Freifeld um mehr als 2 dB abgesenkt werden kann [13].

Weitere Möglichkeiten der Validierung sind Fragebogeninventare, hier haben sich verschiedene bereits etabliert, die wie z. B. das Abbreviated Profile of Hearing Aid Benefit (APHAB) [3, 24, 25]. Beim APHAB werden 24 Fragen zu verschiedenen Hörsituationen in Ruhe, im Störgeräusch, in halligen Räumen und zu unangenehmen Geräuschen gestellt. Die Ergebnisse können ohne und mit Hörgeräten ausgewertet werden, und der entsprechende Gewinn wird notiert. Ein weiterer häufig angewendeter Fragebogen ist das International Outcome Inventory for Hearing Aids (IOI-HA), ein sehr kurzer Fragebogen mit 7 Items zu verschiedenen Aspekten des täglichen Lebens, so auch zur Lebensqualität, Zufriedenheit und noch bestehenden Einschränkungen in der Teilhabe [4]. Ebenfalls häufig angewendet wird der Fragebogen Speech, Spatial and Qualities of Hearing Scale, der fast 50 Fragen zum Sprachverstehen, dem räumlichen Hören und zur Klangqualität mithilfe einer visuellen Analogskala von 0–10 stellt [12].

**Abb. 8:**
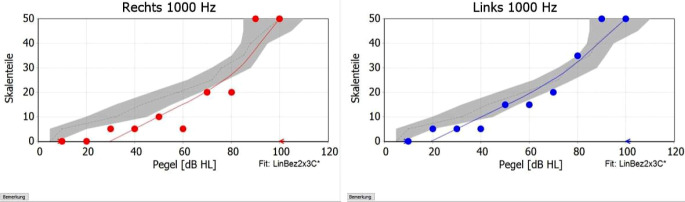
Beispiel für eine Lautheitsskalierung. Abspielen verschieden lauten Terzbandrauschens (Mittenfrequenz hier 1000 Hz), entweder über Kopfhörer oder im Freifeld. Angabe der Testperson über empfundene Lautheit des Rauschens (sehr leise, leise, mittel, laut, sehr laut, zu laut, ggf. mit Zwischenstufen). Ziel: mit der Hörsystemversorgung im *grauen Bereich* des Normalhörenden zum Erreichen einer Lautheitsnormalisierung zu liegen

Ein weiterer Aspekt der Validierung ist in zunehmendem Maße die Messung der Höranstrengung. Gerade Menschen mit einer beginnenden, geringgradigen Schwerhörigkeit profitieren v. a. hinsichtlich der Anstrengung [1]. Inzwischen wurden Messmethoden entwickelt, um die Höranstrengung, z. B. während der Testung eines Sprachtests im Störgeräusch zu messen [18]. Auch die kategoriale Lautheitsskalierung wird gemessen, ein psychoakustisches Messverfahren, bei dem die Testperson die subjektiv empfundene Lautstärke (d. h. Lautheit) der sowohl in Lautstärke als auch Frequenz randomisiert dargebotenen Signale mithilfe einer Skala bewertet – von „nicht gehört“ bis „unangenehm laut“ [14]. Die verbalen Kategorien werden in Zahlenwerte von 0 (nicht gehört) bis 50 (unangenehm laut) übertragen, um dann als Funktion des Pegels grafisch dargestellt zu werden. Damit werden die Hörsysteme lautheitsbasiert angepasst und so eine Normalisierung der Lautheitsempfindung erreicht (Abb. 8).

## Fehlerquellen im Rahmen der Anpassung

Eine häufige Fehlerquelle ist die mangelhafte akustische Ankopplung. Es ist auf einen guten Sitz der Otoplastik zu achten und, um Direktschall zu vermeiden, eine möglichst geschlossene Otoplastik zu wählen (so geschlossen wie möglich, so offen wie nötig). Darüber hinaus sind die Bauform und die Verstärkung so zu wählen, dass eine ausreichende Verstärkung (mit 10–15 dB Reserve) gewährleistet ist. Ohne eine Verifikationsmessung liefert die Validierung u. U. nicht den Anhaltspunkt für eine optimale Versorgung, nur mit dem Wissen, dass die Verstärkung dem Hörverlust angemessen ist, zeigt die Validierung das optimale Hörhilfenergebnis. Die Darstellung in der Hersteller-Fitting-Software ist lediglich eine Berechnung und ersetzt die Verifikation mit einem unabhängigen System nicht.

## Fazit für die Praxis

- Die Hörsystemversorgung ist eine interdisziplinäre Aufgabe, insbesondere zwischen der Hals-Nasen-Ohren-Heilkunde und der Hörakustik.
- Grundlage der Anpassung ist eine ausführliche Diagnostik und Differenzialdiagnostik sowie die Bedarfsanalyse.
- Es ist die medizinische Verantwortung eine entsprechende Therapie zu wählen.
- Kommt eine konservative Hörgeräteversorgung infrage, sollte eine individuelle Otoplastik zur bestmöglichen akustischen Ankopplung Standard sein, ebenso wie Messungen zur Verifikation und Validierung der Hörsystemeinstellung, wobei die Messungen des Hörerfolgs an die Bedürfnisse der Versorgten angepasst sein sollten.
- Im Rahmen einer qualitätsbasierten Hörsystemversorgung ist es daher zum einen notwendig, evidenzbasierte Standards festzulegen, und die Prozessverantwortung, insbesondere die qualitativ hochwertige Überprüfung der Versorgung, in der Hand der HNO-Medizin und Audiologie zu geben, v. a. auch, um die Grenzen der Anpassung frühzeitig festzustellen und eine weiterführende Therapie, wie z. B. die Versorgung mit einem Cochleaimplantat zu initiieren.
